# Impact of Coronavirus Outbreak on NO_2_ Pollution Assessed Using TROPOMI and OMI Observations

**DOI:** 10.1029/2020GL087978

**Published:** 2020-06-05

**Authors:** M. Bauwens, S. Compernolle, T. Stavrakou, J.‐F. Müller, J. van Gent, H. Eskes, P. F. Levelt, R. van der A, J. P. Veefkind, J. Vlietinck, H. Yu, C. Zehner

**Affiliations:** ^1^ Royal Belgian Institute for Space Aeronomy (BIRA‐IASB) Brussels Belgium; ^2^ Royal Netherlands Meteorological Institute (KNMI) De Bilt The Netherlands; ^3^ Department of Geoscience and Remote Sensing Delft University of Technology (TU Delft) Delft The Netherlands; ^4^ ESA/ESRIN Frascati Italy

**Keywords:** air quality, satellite NO_2_, coronavirus outbreak, lockdown, emissions

## Abstract

Spaceborne NO_2_ column observations from two high‐resolution instruments, Tropospheric Monitoring Instrument (TROPOMI) on board Sentinel‐5 Precursor and Ozone Monitoring Instrument (OMI) on Aura, reveal unprecedented NO_2_ decreases over China, South Korea, western Europe, and the United States as a result of public health measures enforced to contain the coronavirus disease outbreak (Covid‐19) in January–April 2020. The average NO_2_ column drop over all Chinese cities amounts to −40% relative to the same period in 2019 and reaches up to a factor of ~2 at heavily hit cities, for example, Wuhan, Jinan, while the decreases in western Europe and the United States are also significant (−20% to −38%). In contrast with this, although Iran is also strongly affected by the disease, the observations do not show evidence of lower emissions, reflecting more limited health measures.

## Key Points

- Satellite NO_2_ data show substantial decreases by 40% on average over Chinese cities due to lockdown measures against the coronavirus outbreak
- Western Europe and United States display robust NO_2_ decreases in 2020, 20–38% relative to the same period in 2019
- Satellite NO_2_ data above Iran, a region strongly affected by coronavirus, do not show clear evidence of lower emissions

## 1 Introduction

Nitrogen oxides (NO_x_ = NO_2_ + NO) are among the main drivers in air quality degradation in urban/industrialized centers, due to their role as catalysts of tropospheric ozone formation, and as precursors of secondary inorganic aerosols, with consequences for climate and human health (Atkinson et al., 2018; Lelieveld et al., 2015; Myhre et al., 2013). The anthropogenic source of NO_x_, primarily originating in fuel combustion, accounts for about 65% of the global total NO_x_ emission, the rest being due to emissions from vegetation fires, lightning, and soils. Due to their link with human activities, NO_x_ atmospheric levels over cities show a weekly cycle with clear minima during the official rest days in most countries (Beirle et al., 2003); important reductions were also reported during public holidays, like the Chinese New Year (Tan et al., 2009). Due to their adverse health effects, the emissions of NO_x_ and other pollutants are regulated in many countries. Long‐term records of satellite observations of NO_2_ columns have been previously used to assess the effectiveness of long‐term abatement strategies (Duncan et al., 2016; De Foy et al., 2016; van der A et al., 2017) and the effects of economic recession (Castellanos & Boersma, 2012). Moreover, satellite observations complemented by in situ measurements have been used to determine the impact on air quality of short‐term emission regulations during specific events, like the 2008 Olympic Games in Beijing (Guo et al., 2013; Mijling et al., 2009), the 2014 Youth Olympic Games in Nanjing (Ding et al., 2015), the 2010 World Expo in Shanghai (Hao et al., 2011), and the 2014 Asia‐Pacific Economic Cooperation summit in Beijing (Huang et al., 2015; Liu et al., 2016).

The ongoing global outbreak of coronavirus disease (Covid‐19), declared as a public health emergency of international concern by the World Health Organization (2020a), led to unprecedented public health responses in many countries around the world including travel restrictions, curfews, and quarantines. The most drastic and consequential quarantines were those of Hubei province in China (Griffiths & Woodyatt, 2020) and Italy (Horowitz, 2020). Their enforcement, combined with measures in other countries, and voluntary limitations of activity (Kim, 2020) result in sweeping disruptions of social and economic activities and even risk of global recession (Leggett, 2020).

In this study, we investigate the impacts of activity reductions resulting from the spread of Covid‐19 on NO_2_ levels in China, South Korea, Italy, Spain, France, Germany, Iran, and the United States, all major epicenters of the outbreak. To that aim, we use NO_2_ column data from two high‐resolution nadir‐viewing satellite sensors, the Tropospheric Monitoring Instrument (TROPOMI), single payload of the Sentinel‐5 Precursor launched in October 2017 (Veefkind et al., 2012), and the Ozone Monitoring Instrument (OMI, Levelt et al., 2006) launched in 2004 on the Aura platform. Both sensors have an overpass local time of ~13:40 and provide daily global coverage at resolutions of 5.5 × 3.5 km^2^ (TROPOMI) and 13 × 24 km^2^ (OMI).

## 2 Satellite Observations and Processing

The OMI QA4ECV NO_2_ data set is based on revised spectral fitting features accounting for improved absorption cross sections, instrument calibration, and surface effects (Boersma et al., 2018; Zara et al., 2018). The data are processed according to the data quality recommendations (Boersma, Eskes, et al., 2017) and were validated against ground measurements (Compernolle et al., 2020). The TROPOMI NO_2_ data benefit from the developments of the OMI QA4ECV retrieval (van Geffenet al., 2019). In this work we use TROPOMI global daily gridded data at 0.05° × 0.05° derived from the near‐real‐time operational product (van Geffen, et al., 2019, 2020), obtained via the Copernicus open data access hub (https://s5phub.copernicus.eu).

To generate time series of NO_2_ columns over specific locations, we first select pixels from an overpass area, defined by a 100‐km radius around the location of interest. For TROPOMI, we use data for which the quality assurance value is higher than 0.5 and the cloud fraction within the NO_2_ retrieval window is below 40%. For OMI, we follow the recommended quality filter criteria (Boersma, et al., 2017), slightly adapted by Compernolle et al. (2020), as well as the cloud fraction upper limit of 40%. The recommended cloud fraction filtering (Boersma et al., 2018; van Geffen et al., 2019, 2020) is actually more strict but deteriorates the statistics, especially above aerosol‐polluted regions. Moreover, focusing only on near‐cloud‐free scenes could lead to a negative sampling bias (Compernolle et al., 2020) as NO_2_ polluted scenes tend to be excluded. Per overpass, the remaining pixels are averaged arithmetically. 14‐day rolling means are calculated and data from 2020 are compared with the previous year (TROPOMI) or the previous 15 years (OMI), in order to distinguish yearly returning patterns (like the Chinese New Year holiday) from the measures taken against the spread of coronavirus and to link these measures with the observed NO_2_ levels. The most recent observation date included in this study is 13 April 2020. We have tested an alternative data selection adopting a 50‐km radius (instead of 100 km) to assess the robustness of the NO_2_ reductions.

TROPOMI and OMI single‐pixel uncertainties in the winter period are typically ~40–60% of the total column value (value based on Wuhan and Milan overpasses). However, in this work we apply temporal or spatial averaging, which will cancel out part of the error (its random component), while the systematic error component is persistent. When we report relative differences between 2019 and 2020, a major part of this systematic error is expected to cancel out.

The systematic absolute difference between TROPOMI and OMI NO_2_ columns (Figure 2 and Figure S2 in the supporting information) stems mainly from the use of different cloud pressure products, O_2_‐O_2_ cloud retrievals used for OMI (Veefkind et al., 2016) and FRESCO retrievals in the O_2_‐A band used for TROPOMI. In the current implementation of FRESCO O_2_‐A in the TROPOMI processor, the algorithm is found to overestimate the pressure for near‐surface clouds or thick aerosol layers. Especially in conditions with moderate to high aerosol pollution levels, the algorithm produces NO_2_ columns that are on average 10–12% lower than OMI in Europe and the United States and up to 20% lower in eastern China (Eskes et al., 2020). Note, however, that most of this OMI‐TROPOMI difference is expected to cancel out when we calculate column ratios between 2019 and 2020.

## 3 Results

### 3.1 China

A lockdown was enforced in Wuhan and other cities of the Hubei province on 23 January (Table S1 and Figure S1). On 2–4 February, it was extended to several cities in other provinces (Table 1). Analyzing the impacts of the lockdown is complicated by the coincidence of Chinese New Year holidays (24 January to 2 February 2020) with the initial phase of the Hubei lockdown. The NO_2_ reduction during the New Year holiday period is a yearly returning phenomenon (Tan et al., 2009). In addition, NO_2_ abundances exhibit a pronounced seasonal cycle (Shah et al., 2020) with highest values during the winter when the NO_x_ lifetime is longest due to low solar irradiances and low specific humidity.

**Table 1:** NO_2_ Column Reduction Observed During the Lockdown Period, Starting on the Reference Date and Lasting 21 Days, Except for Iran Where It Lasts 17 Days, Relative to the Same Period in 2019

City	Lat	Lon	Reference date	TROPOMI	OMI
Beijing	39.9	116.4	11‐Feb‐20	−25(±10)%	−33(±10)%
Chengdu	30.7	104.1	11‐Feb‐20	−19(±21)%	−10(±27)%
Chonqing	30.7	104.1	11‐Feb‐20	−43(±14)%	−11(±32)%
Dalian	38.9	121.6	11‐Feb‐20	−45(±8)%	−18(±16)%
Dongguan	23.0	113.7	11‐Feb‐20	−14(±16)%	−36(±11)%
Foshan	38.9	121.6	11‐Feb‐20	−34(±12)%	−51(±9)%
Guangzhou	23.1	113.3	11‐Feb‐20	−30(±14)%	−56(±8)%
Jinan	36.7	117.0	11‐Feb‐20	−69(±4)%	−63(±5)%
Nanjing	32.1	118.8	11‐Feb‐20	−49(±8)%	−57(±9)%
Qingdao	36.1	120.4	11‐Feb‐20	−54(±6)%	−43(±11)%
Shanghai	31.2	121.5	11‐Feb‐20	−11(±15)%	−29(±14)%
Shenyang	41.8	123.4	11‐Feb‐20	−52(±7)%	−29(±12)%
Tianjin	39.1	117.2	11‐Feb‐20	−46(±8)%	−37(±10)%
Wuhan	30.6	114.3	11‐Feb‐20	−43(±14)%	−57(±14)%
Xian	34.3	109.0	11‐Feb‐20	−56(±9)%	−57(±10)%
Zhengzhou	34.8	113.6	11‐Feb‐20	−53(±7)%	−64(±6)%
Milan	36.7	117	23‐Feb‐20	−38(±10)%	−24(±13)%
Venice	45.4	12.3	23‐Feb‐20	−33(±9)%	−33(±11)%
Madrid	40.4	−3.7	15‐Mar‐20	−29(±12)%	−21(±21)%
Barcelona	41.4	2.2	15‐Mar‐20	−32(±12)%	−31(±20)%
Paris	48.8	2.4	17‐Mar‐20	−28(±10)%	−28(±12)%
Brussels	50.9	4.4	17‐Mar‐20	−18(±11)%	−22(±11)%
Frankfurt	50.1	8.7	23‐Mar‐20	−21 (±11)%	−23(±13)%
Hamburg	53.6	10.0	23‐Mar‐20	−19(±12)%	−21(±15)%
Tehran	35.7	51.4	4‐Mar‐20	−27(±20)%	18(±19)%
Isfahan	32.7	51.7	4‐Mar‐20	37(±16)%	19(±19)%
Daegu	35.9	128.6	23‐Feb‐20	−24(±10)%	−34(±13)%
Seoul	37.6	127.0	23‐Feb‐20	−43(±7)%	−30(±10)%
New York	40.7	−73.9	24‐Mar‐20	−28(±11)%	−31(±14)%
Washington	38.9	−77.0	24‐Mar‐20	−21(±13)%	−12(±25)%
Philadelphia	39.9	−75.2	24‐Mar‐20	−24(±11)%	−11(±21)%
Chicago	41.9	−87.6	24‐Mar‐20	−19(±12)%	3(±25)%
Detroit	42.3	−83.0	24‐Mar‐20	−21(±12)%	−23(±21)%

*Note*. The averages are calculated using data within a 100 km radius around the city centers. The coordinates (latitude north and longitude east) are given. The lockdown period for China and Iran was chosen in order to eliminate interference of the national holidays. The uncertainties given within brackets are standard errors calculated from the retrieval uncertainties and accounting for the number of days with valid data.

**Figure 1:**
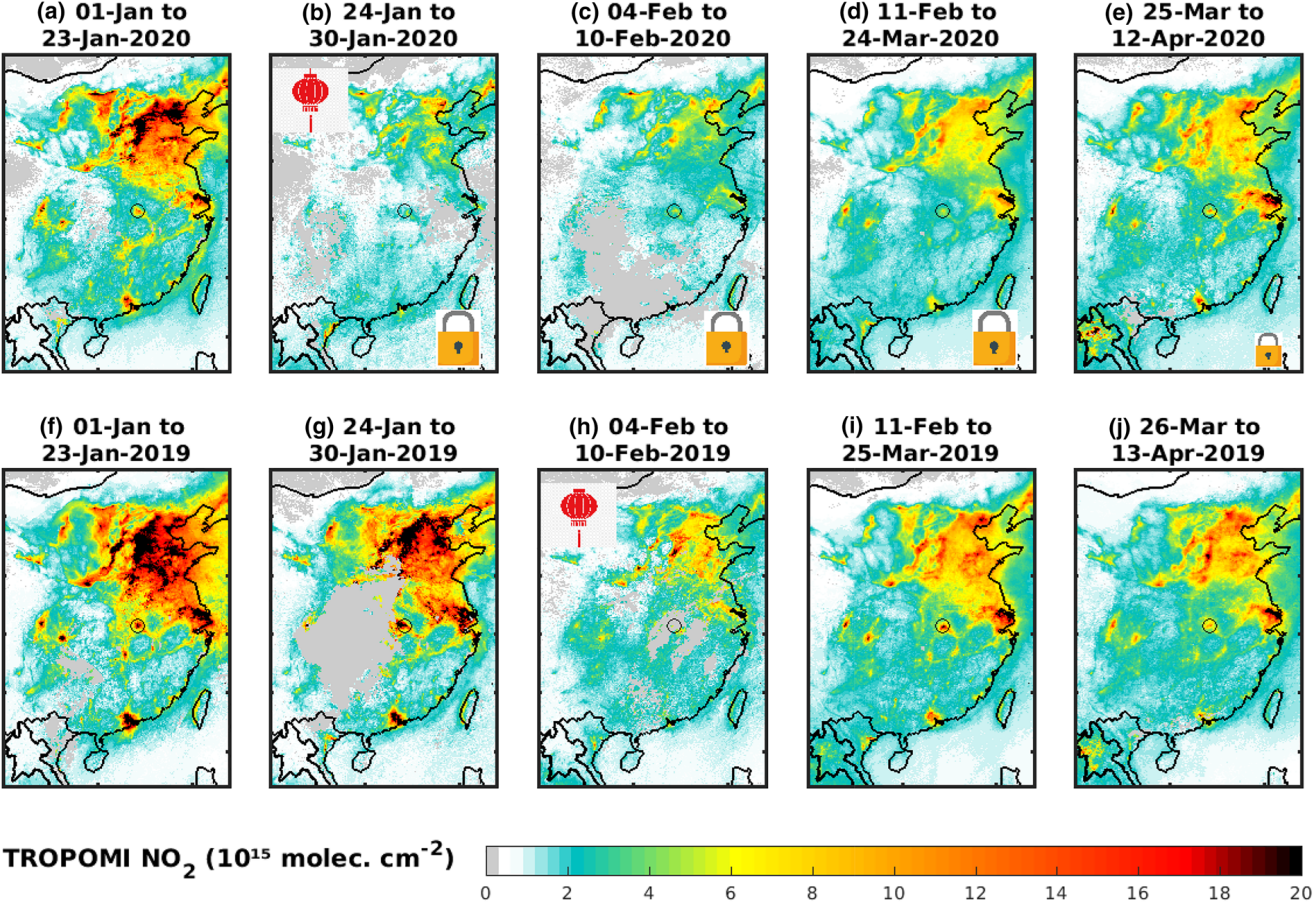
TROPOMI NO_2_ columns over China (a) before and (b–e) after the lockdowns (shown by padlocks) due to Covid‐19. For comparison, columns over the same time periods are shown for 2019. The week of Chinese new year holiday is indicated by the red lantern shown inset panels (b) and (h). The New Year holiday covers 4–10 February in 2019 and 24–30 January in 2020 (exceptionally extended to 2 February because of Covid‐19). Partial loosening of the restrictions is suggested by the smaller padlock in panel (e). Gray areas indicate no valid data.

**Figure 2:**
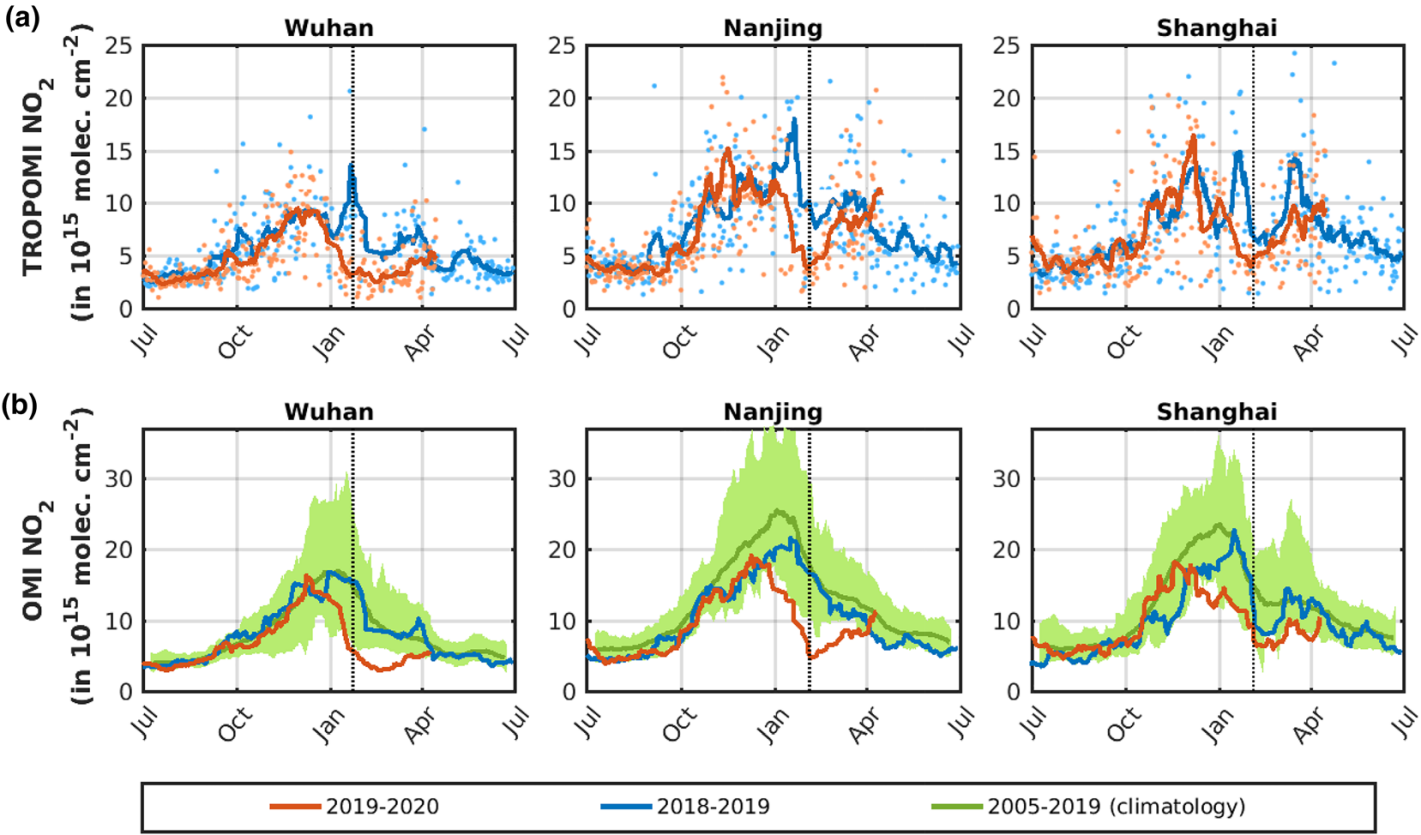
Spaceborne NO_2_ columns within a 100 km radius around Wuhan, Nanjing, and Shanghai (blue symbols for 2018–2019 and red symbols for 2019–2020) and 14‐day running averages (colored lines). (a) TROPOMI. (b) OMI, including climatological columns (2005–2019) and their range (green).

Figure 1 presents time‐averaged NO_2_ columns over China over successive periods in 2020 (a–e) and 2019 (f–j), with indication of the Chinese New Year holiday and of the lockdown. In 2019, TROPOMI observations point to a strong decrease in NO_2_ columns during the New Year holiday (4–10 February), reflecting mainly lower emissions during that period. The NO_2_ columns recover afterward (11 February to 25 March 2019) but to levels significantly lower compared to the January values, primarily due to NO_x_ lifetime changes. The seasonality of NO_2_ columns at Chinese cities is well illustrated in Figures 2 and S2, displaying the evolution of NO_2_ columns over Chinese cities between July 2019 and June 2020, as seen by TROPOMI (upper row) and OMI (lower row). Besides the strong short‐term variability (due to a combination of meteorological variability and large random observational errors), the wintertime maximum is evident at all cities, especially when considering the climatological OMI values averaged over 2005–2019 (green line in the lower panel of Figures 2 and S2). For example, at Wuhan and Nanjing, the columns decrease by about a factor of 2 between January and early March.

In 2020, the coincidence of the initial lockdown phase with the New Year holidays (24 January to 2 February) causes a steep drop of NO_2_ columns, reaching a factor of 2 or more at most cities (Figures 1b, 2, and S2). After the holidays, however, the strong NO_2_ reduction persists for several weeks, after which the columns recover progressively, reflecting the return of economic activities and emissions. As seen in Figure 2, the NO_2_ decreases are very sharp in Wuhan and Nanjing. In these two cities where strict lockdowns were enforced, the columns in the lockdown period are not only much lower than during the same period of the previous year but also lower than in all previous 15 years of OMI observations. According to TROPOMI data, whereas NO_2_ levels stagnated in Wuhan until mid‐March, they recovered more rapidly in other cities and returned to near‐global levels in early April. Similar behavior is found at most other Chinese cities like Shenzhen, Qingdao, Zhengzhou, and Xian (Figure S2).

A quantitative estimate of the impact of the lockdown on NO_2_ columns at Chinese cities is given in Table 1. To eliminate the interference of seasonal variation and New Year holidays, the average NO_2_ column between 11 February and 24 March 2020 is compared to the average column in the same period in 2019. The average NO_2_ drop calculated over Chinese cities affected by the lockdown amounts to −40% according to both TROPOMI and OMI (Table 1) and to −45% when the smaller (50 km) radius is adopted for calculating the averages (Figure S3). Stronger reductions of up to −60% are found at the most affected cities, including Wuhan and Xi'an (Figure S3). The decrease is lower for many cities not strongly affected by the lockdown, for example, Beijing (−25% to −40%).

These reductions are comparable with those observed as consequence of temporary stringent emission regulations, for example, in Beijing during the APEC Blue event in November 2014 (−21%) and Parade Blue in August 2015 (−43%) (Liu et al., 2016), and with the column reduction reported during the 2008 Olympic Games in Beijing (−46%, Mijling et al., 2009).

The observed column decreases are due to the decline of traffic emissions, by far the dominant NO_x_ emission source in cities, as well as to decreases in industrial activities and power generation. A significant drop (−50%) in coal‐fired power generation was recorded in the 10 days following the Chinese New Year in 2020 compared to 2019 (Myllyvirta, 2020). This decline was prolonged for 40 days after the New Year and a clear rebound was reported afterward (Figure S4). Substantial decreases were also recorded in industrial activity indicators (e.g., steel production and oil refineries). Moreover, air traffic in China dropped in mid‐February by 80% compared to January 2020 and showed a small recovery in the beginning of March, although still 61% lower than in January (Zara, 2020).

### 3.2 Western Europe

**Figure 3:**
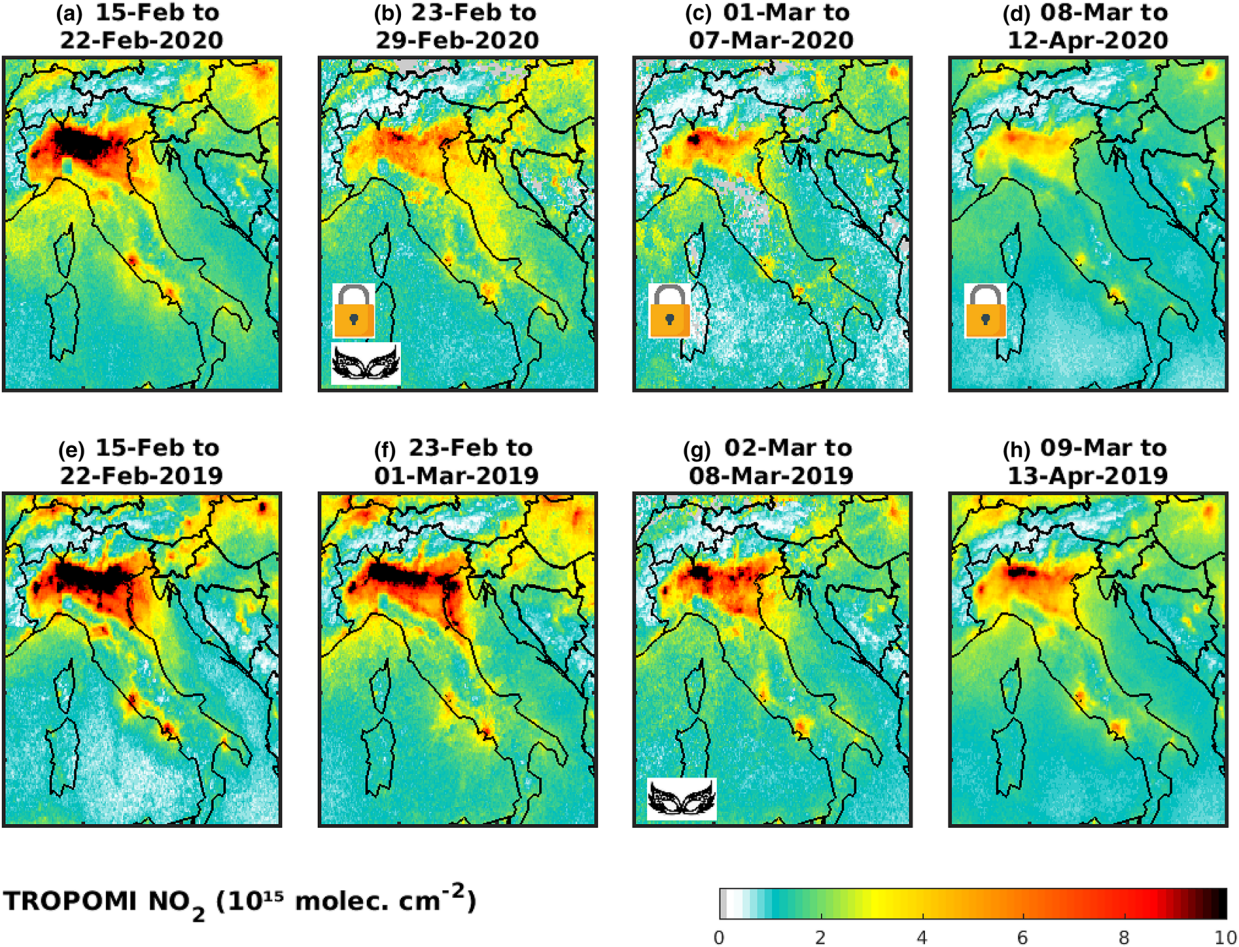
TROPOMI NO_2_ columns over Italy (a) before and (b–d) during the lockdown in 2020 and over the same time periods for 2019. Note that 2020 is a leap year. The week of Carnival holidays (Table S1) is indicated by masks in panels (b) and (g). Gray areas indicate no valid data.

In Italy, the outbreak of Covid‐19 led first to the quarantine of 11 small towns in Lombardy, close to Milan, and in the Veneto province, starting on 23 February. On 4 March, the government ordered the nationwide lockdown of schools and universities. As in the case of China, the analysis has to take into account the (photochemically induced) seasonal variation of NO_2_ columns and the near coincidence of the first lockdown with an important holiday, the Carnival, which took place on 24–25 January 2020 (Table S1). As seen in Figures 3e–3h, seasonal variations and the Carnival of 2019 might explain (part of) the decline of NO_2_ columns measured by TROPOMI over northern Italy in early March 2019, although synoptic meteorological variability might also contribute.

In 2020, a substantial reduction of NO_x_ emissions is apparent in the last week of February, especially in Lombardy and Veneto (comparing Figures 3a and 3b). This reduction, much larger than the corresponding decline in 2019 (panels e and f), is likely mainly due to lower economic activities in response to both the Carnival holidays and the initial measures taken to counter the coronavirus. In March–April, the columns remain very low, not only in comparison with the prelockdown values (comparing panels c and d with a) but also compared to the corresponding period in 2019 (g and h). The NO_2_ levels are unusually low not only in Lombardy and Veneto but also elsewhere in Italy, for example, above Turin and Bologna. The average TROPOMI NO_2_ column during the lockdown period in 2020 is found to be between 38(±10)% and 33(±9)% lower than during the same period in 2019 in Milan and Venice (Table 1). Those reductions outweigh the combined effect of emission regulations and economic recession on OMI NO_2_ columns in Europe over 2004–2010, which was estimated at 20% (Castellanos & Boersma, 2012).

In response to the Covid‐19 infection, other European countries declared a state of emergency and placed their countries under lockdown and stay‐at‐home orders (Table S1). Spain became the most affected country in Europe in number of cases, followed by Italy, France, and Germany (as of 25 April 2020), and lockdown measures were enacted in these countries on 14, 17, and 22 March, respectively. As a result of these measures, a marked NO_2_ column drop (−30%) was observed during the strict lockdowns in Spain and France in comparison with the same period in 2019, whereas more moderate decreases were found in Germany and Belgium (−20%), possibly because the lockdown conditions were less strict in these countries (Figure S5). The average NO_2_ decrease over the European cities of Table 1 is very similar between TROPOMI (−27%) and OMI (−25%).

### 3.3 South Korea

South Korea has been severely affected by the outbreak, although containment measures in these countries were much more limited than in Chinese areas affected by the coronavirus (Table S1). A reduction of economic activities and traffic is nevertheless expected and should be reflected in the satellite NO_2_ measurements. Although the first reports of Covid‐19 in South Korea appeared on 20 January 2020 (World Health Organization, 2020b), the South Korean government declared the highest level of health alert on 23 February and asked to refrain from unnecessary travel. While these measures are mostly on a voluntary basis, they clearly led to emission reduction over South Korean cities such as Seoul and Daegu, where the average NO_2_ columns during the three 3 weeks after the reference date (health alert on February 23) are between −43(±7)% (Seoul) and −24(±10)% (Daegu) lower than during the same period 1 year earlier, based on TROPOMI measurements (Table 1 and Figure S6). It is, however, not excluded that a small fraction of the reduction could be due to meteorological variability (wind, cloud cover, and humidity), which can affect the transport and lifetime of NO_x_. Substantial column reductions during these 4 weeks in 2020 (relative to 2019) are also evident at other large cities including Gwangju (southwest) and Cheongju (center), although not at Busan (south), the second‐largest city (Figure S6). The reduction is also clear when comparing the columns on the weeks before and after the lockdown (comparing Figures S6a and S6b). The columns appeared, however, to recover in late March and early April (panel c), at least in the Seoul and Daegu areas.

### 3.4 Iran

Reports that the virus had reached Iran occurred on 20 February 2020 (World Health Organization, 2020c). Although the Iranian government has ordered the closure of schools and universities on 23 February and imposed a limit on public gatherings on 3 March, these measures are not clearly reflected in the observed NO_2_ signal. Compared to the previous year, the average NO_2_ levels in Tehran and Isfahan were higher in the first weeks after the reference date (23 February) (Table 1). The temporal evolution of the columns over Tehran, the capital city and strongest NO_2_ hot spot (Figure S7), does not indicate significant NO_2_ changes in the period before (1–22 February) and after the lockdown (4–20 March). Note that the temporal variability of TROPOMI NO_2_ columns over Tehran in wintertime is very strong, reflecting to a large extent the unusually large NO_2_ retrieval errors, exceeding 100% over the Tehran agglomeration (Figure S8), although meteorological variability might be an additional contributing factor. A likely explanation for the absence of NO_2_ reduction between 4 and 20 March is that complete lockdowns were not enforced in Iran, as the administration initially ruled out this possibility, while urging people to voluntarily stay at home (Mehdi, 2020). Moreover, Tehran's lockdown was not approved because the government could not provide financial aid to people and businesses. On 28 March, a complete lockdown was imposed (Mehdi, 2020), but stay‐at‐home calls were largely ignored (Wintour, 2020). The sharp NO_2_ column decline from 21 March to 4 April detected in 2019 and 2020 is due to Nowruz, a 2‐week celebration marking the beginning of the Iranian New Year (Figure S7). Compared to the 3‐week period just before the New Year, the columns are reduced by factor of 2–2.5 in Tehran, Isfahan, and Qom in 2020.

### 3.5 United States

**Figure 4:**
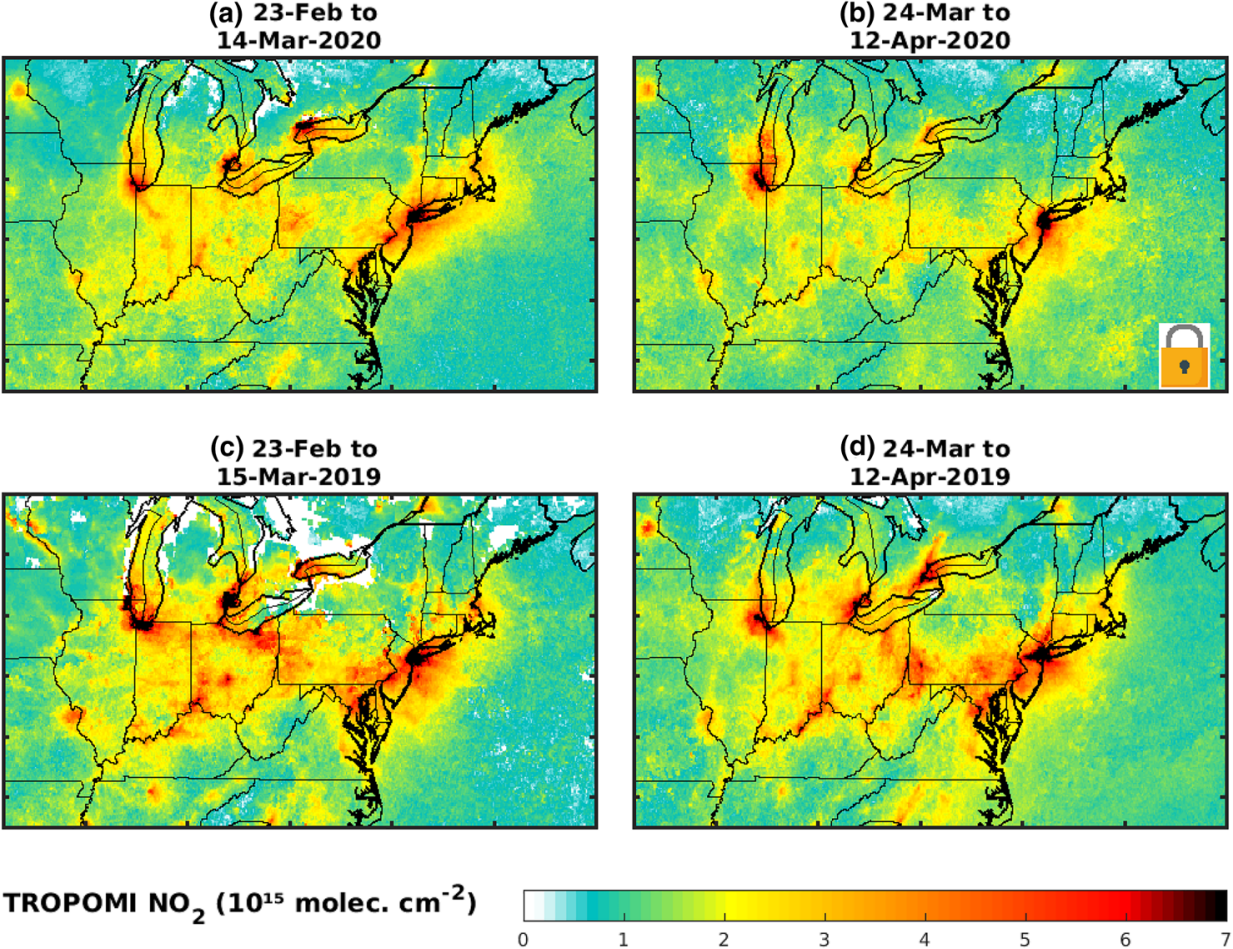
TROPOMI NO_2_ columns over northeastern United States before and after the lockdowns in 2020 (a, b) and for the same periods in 2019 (c, d).

In March 2020, the focus of the Covid‐19 crisis has shifted decisively from Europe to the United States which became the next global epicenter of the crisis with the biggest number of confirmed cases in the world (worldometers.info/coronavirus, as of 25 April 2020). In an attempt to slow down the progression of the infection, a large majority of state and local governments responded by declaring emergency and issuing stay‐at‐home orders, which came into effect at different dates in late March and early April (Secon & Woodward, 2020). Two periods were chosen for the analysis: 23 February to 14 March 2020 (prelockdown) and 24 March to 13 April 2020 with lockdowns in most of the eastern states, for example, Illinois (21 March), Indiana and Ohio (23 March), and New York (22 March), as summarized in Table S1. In the southeastern states, the lockdowns were enacted in early April, and therefore, the NO_2_ time series are too short to allow robust conclusions. These states were therefore not considered in our analysis. Significant reductions are not only observed over major cities of northeastern United States, −28(±11)% in New York and −24(±11)% in Philadelphia according to TROPOMI (Table 1) but also in the rural areas where the decreases are often stronger (up to 40%, Figure 4). To a large extent, the decreased NO_2_ levels can be attributed to the reported road traffic decline in the United States by 42% in 21–27 March, 47% in 28 March to 3 April, and 48% in 4–10 April compared to the control week of 22 February 2020 (Schuman, 2020). For a small part, the NO_2_ decrease is also explained by emission regulations and their impacts on long‐term emission trends, estimated to be of the order of −4%/yr at most between 2005 and 2014 over northeastern U.S. cities (Duncan et al., 2016).

## 4 Conclusions

Exceptional decreases in NO_2_ columns were observed over widespread areas in China, Europe, South Korea, and the United Sates in January–April 2020, based on high‐resolution spaceborne data. Those decreases are evident not only from the comparison of NO_2_ levels before and during the lockdown but also when contrasting the 2020 levels with those during the same period in 2019. The decreases are mainly attributed to the containment measures against the spread of the Covid‐19, which caused sharp reductions in traffic and industrial activities. For a small part, the column reductions could also be due to meteorological variability and to the decline of emissions caused by environmental policy regulations. Model studies will therefore be needed to separate the effects of the enforced health measures from other contributing factors. To this purpose, satellite observations of NO_2_ and other compounds (e.g., CO and aerosol optical depth), complemented by in situ observations, will help interpret the observed decreases and assess the full impacts of these measures on air pollution. At the time of drafting, China has lifted most restrictions, while Europe and United States are moving toward a prudent loosening of the measures. Further studies will be needed to evaluate the effects of the temporary lockdowns on global air quality and climate, and the gradual return to prelockdown periods.

## Supporting information



Supporting Information S1
