# Supplementary material for: Impact of Coronavirus Outbreak on NO2 Pollution Assessed Using TROPOMI and OMI Observations
Source: Geophys Res Lett. 2020 Jun 5;47(11):e2020GL087978. doi: 10.1029/2020GL087978 (PMC7261997; doi:10.1029/2020GL087978)
Supplement: Supplementary file 1 — Supporting Information S1 [file GRL-47-0-s001.docx]

**Supplement for**

**Impact of coronavirus outbreak on NO_2_ pollution assessed using TROPOMI and OMI observations**

**M. Bauwens^1^, S. Compernolle^1^, T. Stavrakou^1^, J.-F. Müller^1^, J. van Gent^1^, H. Eskes^2^, P. F. Levelt^2,3^, R. van der A^2^, J. P. Veefkind^2^, J. Vlietinck^1^, Huan Yu^1^, C. Zehner^4^**

^1^ Royal Belgian Institute for Space Aeronomy (BIRA-IASB), Avenue Ciruclaire 3, 1180 Brussels, Belgium

^2^ Royal Netherlands Meteorological Institute (KNMI), Utrechtseweg 297, 3731 GA De Bilt, The Netherlands

^3^ Delft University of Technology (TU Delft), Department of Geoscience and Remote Sensing, Stevinweg 1, 2628 CN Delft, The Netherlands

^4^ ESA/ESRIN, Frascati, Italy

Corresponding author: Trissevgeni Stavrakou (jenny@aeronomie.be)

**Table S1.** Public health measures taken in different countries to prevent the spread of Covid-19 in January-April 2020. The periods of official holidays in China, Italy, Korea and Iran are also given. Notes: ***^1^*** at the moment of drafting

***^a^*** en.wikipedia.org/wiki/2020_Hubei_lockdowns

***^b^*** en.wikipedia.org/wiki/2019-20_coronavirus_pandemic_in_mainland_China

***^c^*** Lowen, M. (2020), Coronavirus: Quarantine raises virus fears in Northern Italy. *BBC News.* Available from bbc.com/news/world-europe-51628084 (Accessed in 25 April 2020)

***^d^*** Coronavirus: Italy to close all schools as deaths rise. *BBC News*. Available from bbc.com/news/world-europe-51743697 (Accessed in 25 April 2020)

***^e^*** Coronavirus: What are the lockdown measures across Europe? *Deutsche Welle News*. Available from www.dw.com/en/coronavirus-what-are-the-lockdown-measures-across-europe/a-52905137

(Accessed in 25 April 2020)

***^f^*** Kim, S. (2020), How South Korea lost control of its coronavirus outbreak. *The New Yorker*. Available from newyorker.com/news/news-desk/how-south-korea-lost-control-of-its-coronavirus-outbreak (Accessed in 25 April 2020)

***^g^*** Cha, V. and Kim, D. (2020), A timeline of South Korea’s response to Covid-19. *Center for Strategic & Intenational Studies (CSIS).* Available from csis.org/analysis/timeline-south-koreas-response-covid-19 (Accessed in 25 April 2020)

***^h^*** Lee, J. (2020), South Korea advises facilities suspension, experts warn of ‘long battle’ against coronavirus. *Reuters News Agency*. Available from reuters.com/article/us-health-coronavirus-southkorea/south-korea-advises-facilities-suspension-experts-warn-of-long-battle-against-coronavirus-idUSKBN218029 (Accessed in 25 April 2020)

***^i^*** Radio France International (2020), Iran closes schools after fifth coronavirus death. Available from rfi.fr/en/international/20200222-iran-closes-schools-after-fifth-coronavirus-death (Accessed in 25 April 2020)

***^j^*** Kottasovà, I. & Mostaghim, R. (2020), Adviser to Iran’s Supreme Leader dies after contracting coronavirus. *CNN*. Available from edition.cnn.com/2020/03/02/middleeast/iran-coronavirus-supreme-leader-adviser-intl/index.html (Accessed in 25 April 2020)

***^k^*** Mehdi, S. Z. (2020), Covid-19: Divergent views at top delay lockdown in Iran. *Anadolu News Agency*. Available from aa.com.tr/en/health/covid-19-divergent-views-at-top-delay-lockdown-in-iran/1782835 (Accessed in 25 April 2020)

***^l^*** Secon, H. and Woodward, A. (2020) About 95% of Americans have been ordered to stay at home. This maps shows which cities and states are under lockdown. *Business Insider*. Available from businessinsider.com/us-map-stay-at-home-orders-lockdowns-2020-3?r=US&IR=T (Accessed in 25 April 2020)

| Date | Location | Measures against Covid-19 |
| --- | --- | --- |
| 23 January 2020, ongoing***^1^*** | Wuhan and other 15 cities of Hubei province | Lockdown on Wuhan and other cities in Hubei province, suspension of all public transport, control of movement***^a^*** |
| 1 February 2020 | Wenzhou (Zhejiang) | Curfew law***^b^*** |
| 4 February 2020 | Hangzhou, Ningbo (Zhejiang)  Zhengzhou (Henan)  Linyi (Shandong)  Harbin (Heilongjiang)  Nanjing, Xuzhou (Jiangsu) Fuzhou (Fujian) | Lockdown***^b^*** |
| 23 February 2020, ongoing***^1^*** | Northern Italy | Lockdown of a small cluster of towns, strict quarantine restrictions close to Milan and Veneto***^c^*** |
| 4 March 2020, ongoing***^1^*** | Italy | Lockdown of schools and universities nationwide***^d,e^*** |
| 14 March 2020, ongoing***^1^*** | Spain | Stay-at-home order, closure of all non-essential shops***^e^*** |
| 17 March 2020, ongoing***^1^*** | France | Nationwide lockdown, stay-at-home order***^e^*** |
| 22 March 2020, ongoing***^1^*** | Germany | Strict social distancing measures, public gatherings banned, closure of all non-essential shops***^e^*** |
| 23 February 2020 | South Korea | Infectious disease alert rises from level 4 (highest category), Voluntary self-quarantine for 2 weeks of affected cities and provinces, no public gatherings, movements not restricted***^f,g^*** |
| 22 March 2020 | South Korea | Enhanced social distancing, closure of facilities***^g,h^*** |
| 22 February 2020 | Iran | Closure of schools and universities and cultural centers in Qom and Tehran***^i^*** |
| 3 March 2020 | Iran | Public gatherings, including Friday prayers cancelled, schools and universities closed nationwide***^j^*** |
| 28 March 2020 | Iran | Strict lockdown, enforcement of social distancing, banned inter-city travel***^k^*** |
| 19 March 2020 | California | Stay-at-home orders, state-wide lockdown***^l^*** |
| 21 March 2020 | Illinois |  |
| 22 March 2020 | New York |  |
| 23-31 March 2020 | Oregon, Washington, New Mexico, Arizona, Minnesota, Wisconsin, Michigan, Indiana, Ohio, Colorado, Kansas, Kentucky, Tennessee, Massachusetts, Louisiana, North Carolina |  |
| 1-7 April 2020 | Nevada, Texas, Mississippi, Alabama, Georgia, Florida, Missouri, Pennsylvania, Maine, South Carolina |  |
| **Date** | **Country** | **Official holidays** |
| 4-10 February 2019  24 January – 2 February 2020 | China | Chinese New Year official public holiday |
| 8-9 March 2019 (Lombardy)  4-6 March 2019 (Veneto)  24-25 February 2020 | Italy | Carnival Holidays |
| 4-6 February 2019  24-26 January 2020 | Korea | Korean New Year official public holiday |
| 21 March-3 April 2019  20 March-4 April 2020 | Iran | Nowruz Holiday |

**
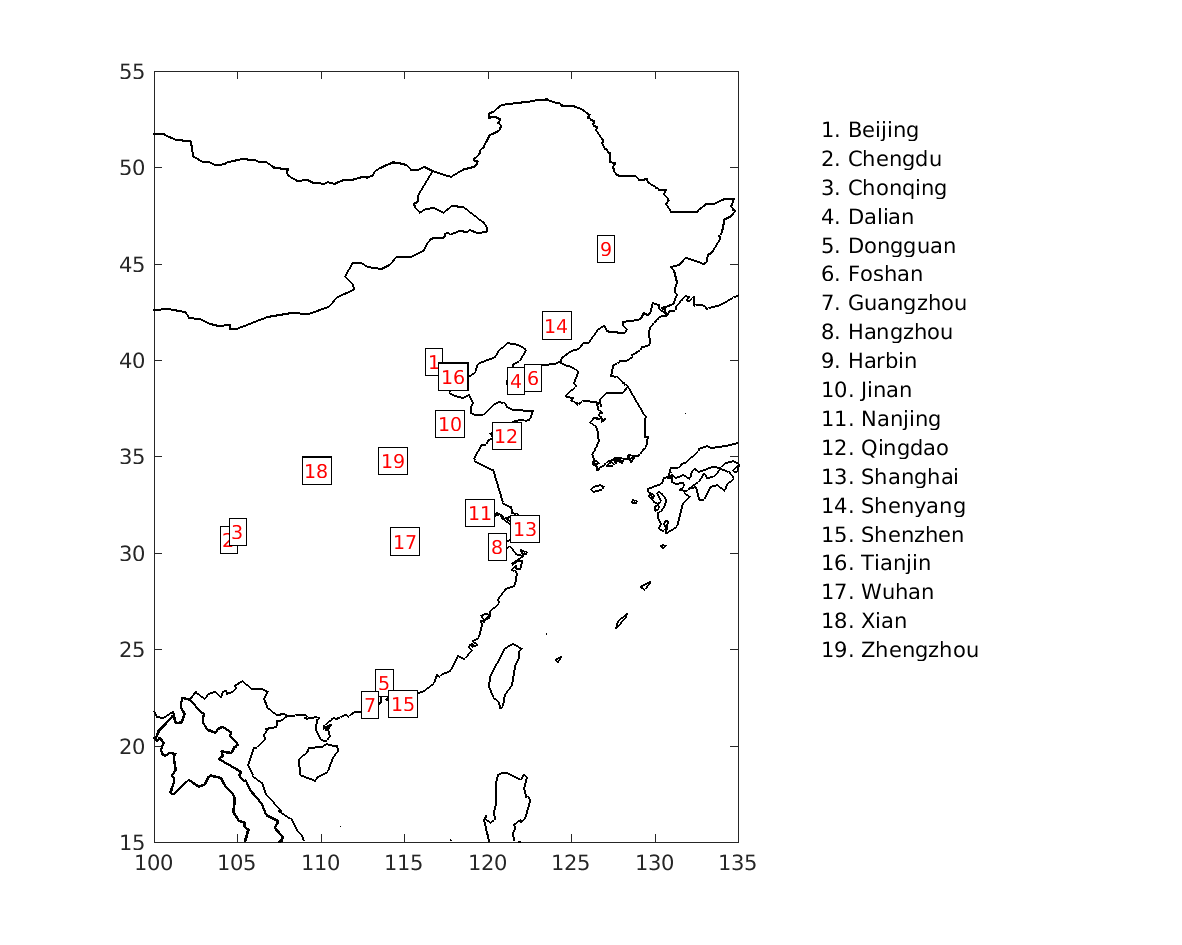
**

**Figure S1.** Locations of the studied Chinese cities.

**
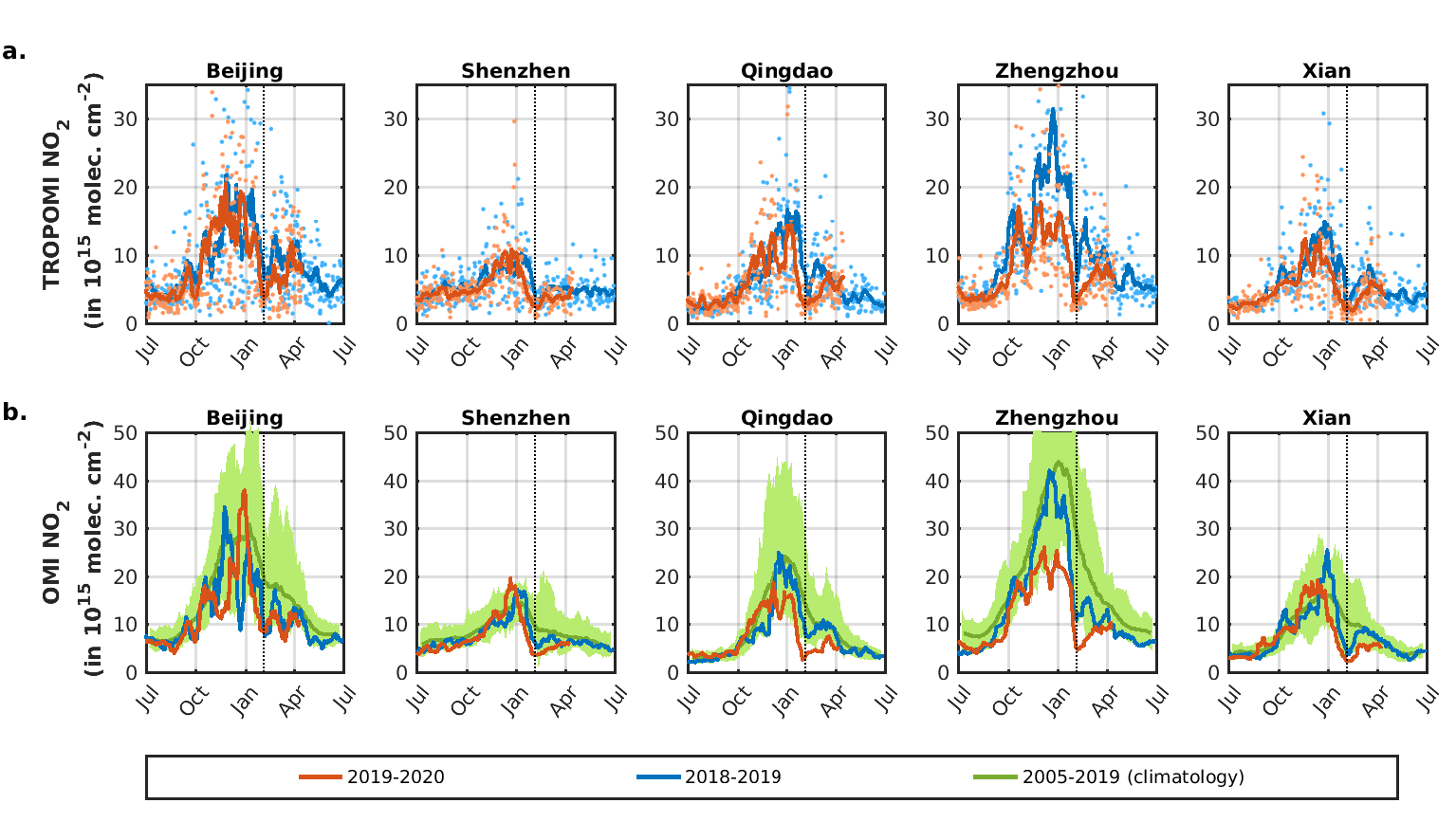
**

**Figure S2.** Same as Figure 2, for five Chinese cities.


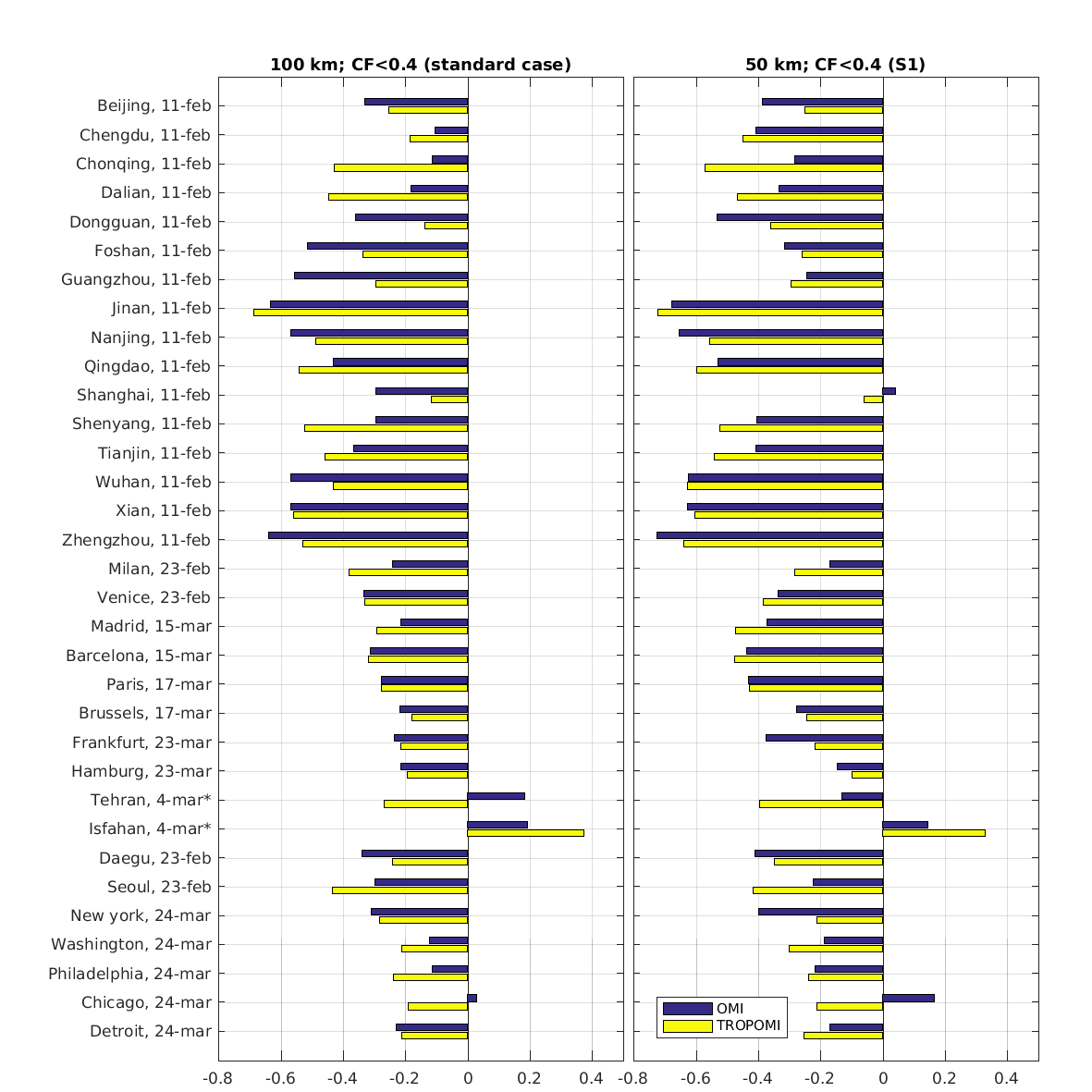


**Figure S3.** Histograms of the NO_2_ column reductions from TROPOMI (yellow) and OMI (dark blue) for 4 different data analyses during the lockdown period (defined as in Table 1), relative to the same period in 2019. The two analyses consider data with cloud fraction lower than 40% and a radius of 100 km (standard case, Table 1), or 50 km around the city centers (S1). The average NO_2_ decrease calculated for all cities according to TROPOMI and OMI is -31%, -29% for the standard case, and -37%, -39% for S1.


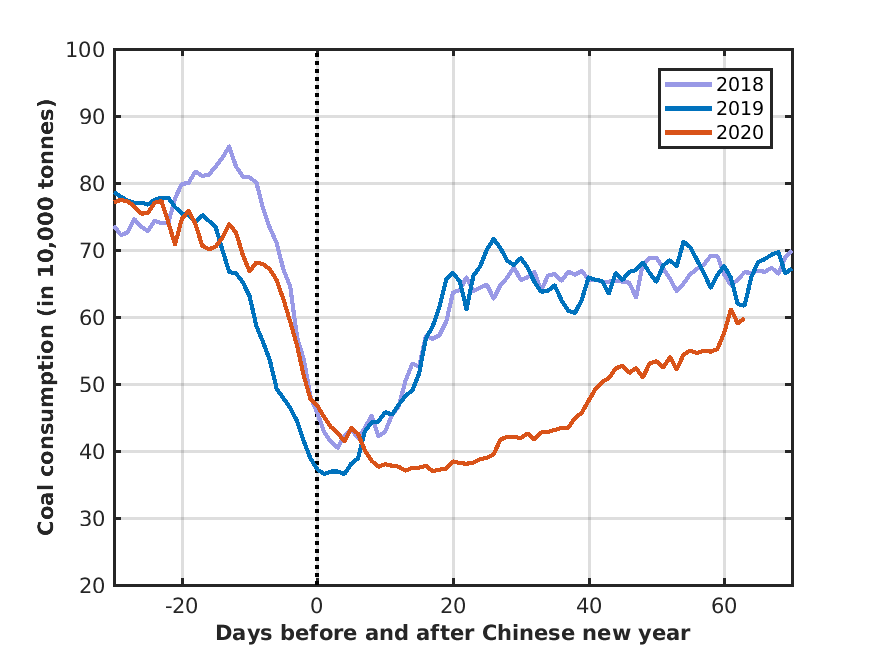


**Figure S4.** Daily coal consumption (in 10,000 tonnes) data at six generating companies in China. Source: Carbonbrief (Myllyvirta, 2020)**.
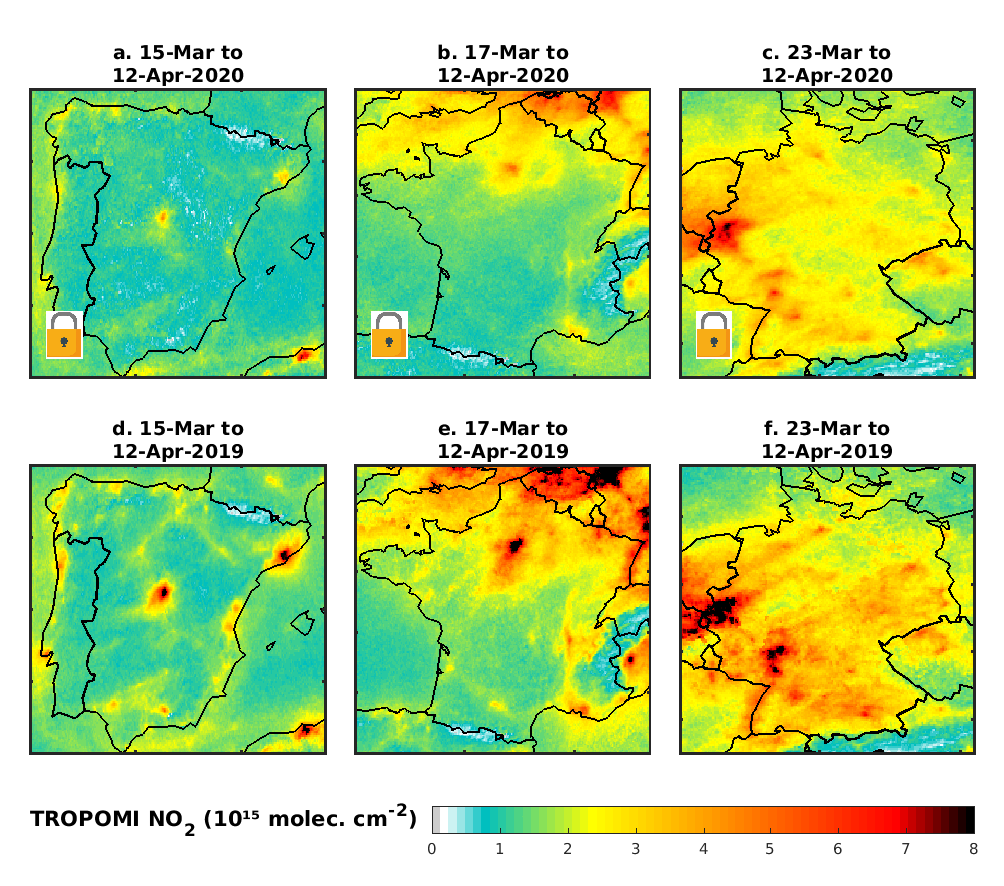
**

**Figure S5.** TROPOMI NO_2_ columns over Spain (a, d), France (b, e) and Germany (c, f) during the lockdown periods in 2020 (upper panels) and 2019 (lower panels).

**
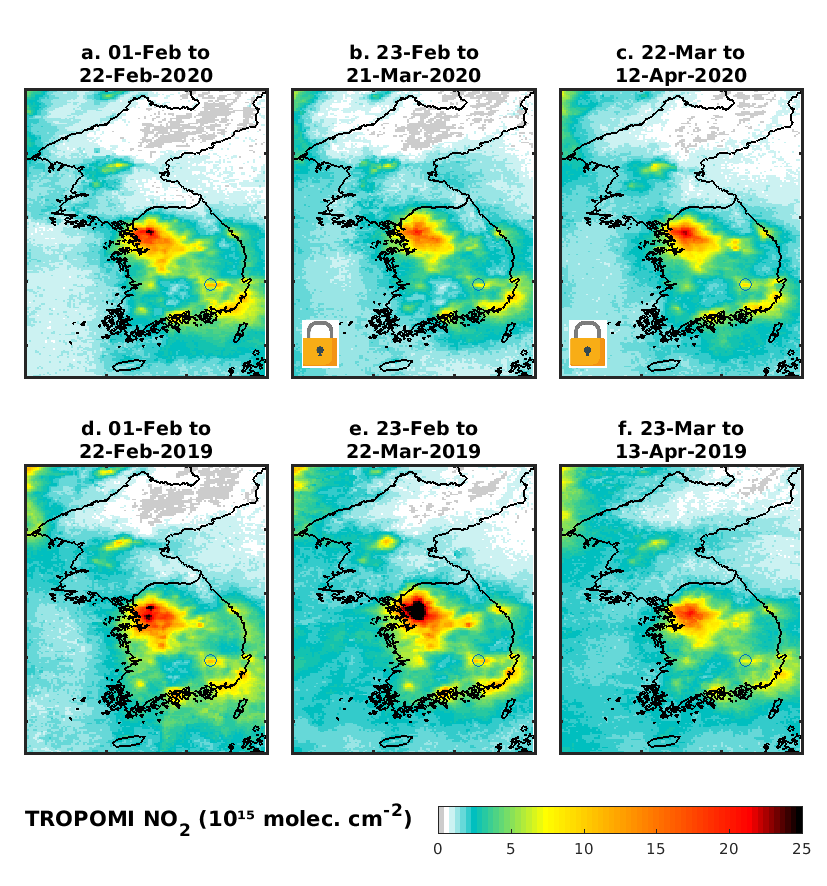
**

**Figure S6.** TROPOMI NO_2_ columns over the Korean Peninsula in 2020 (a-c) and 2019 (d-f). The city of Daegu is indicated by an open circle.

**
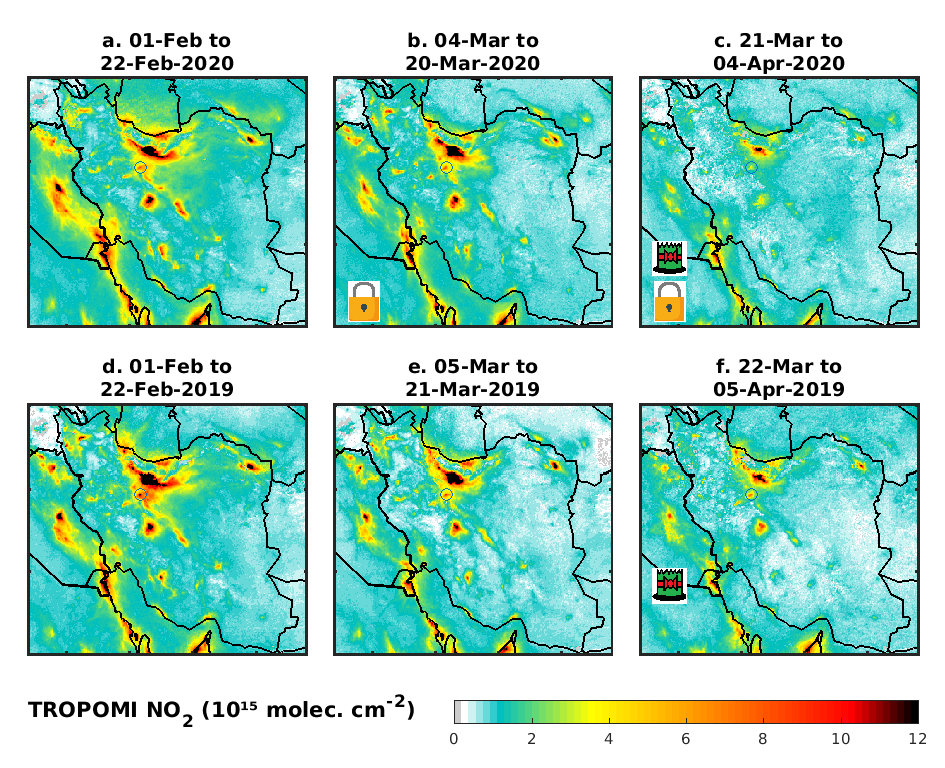
**

**Figure S7.** TROPOMI NO_2_ columns over Iran in 2020 (a-c) and 2019 (d-f). The sprouted wheat ‘sabzeh’ inset indicates Nowruz, a two-week celebration marking the beginning of the New Year in the Iranian cadendar.


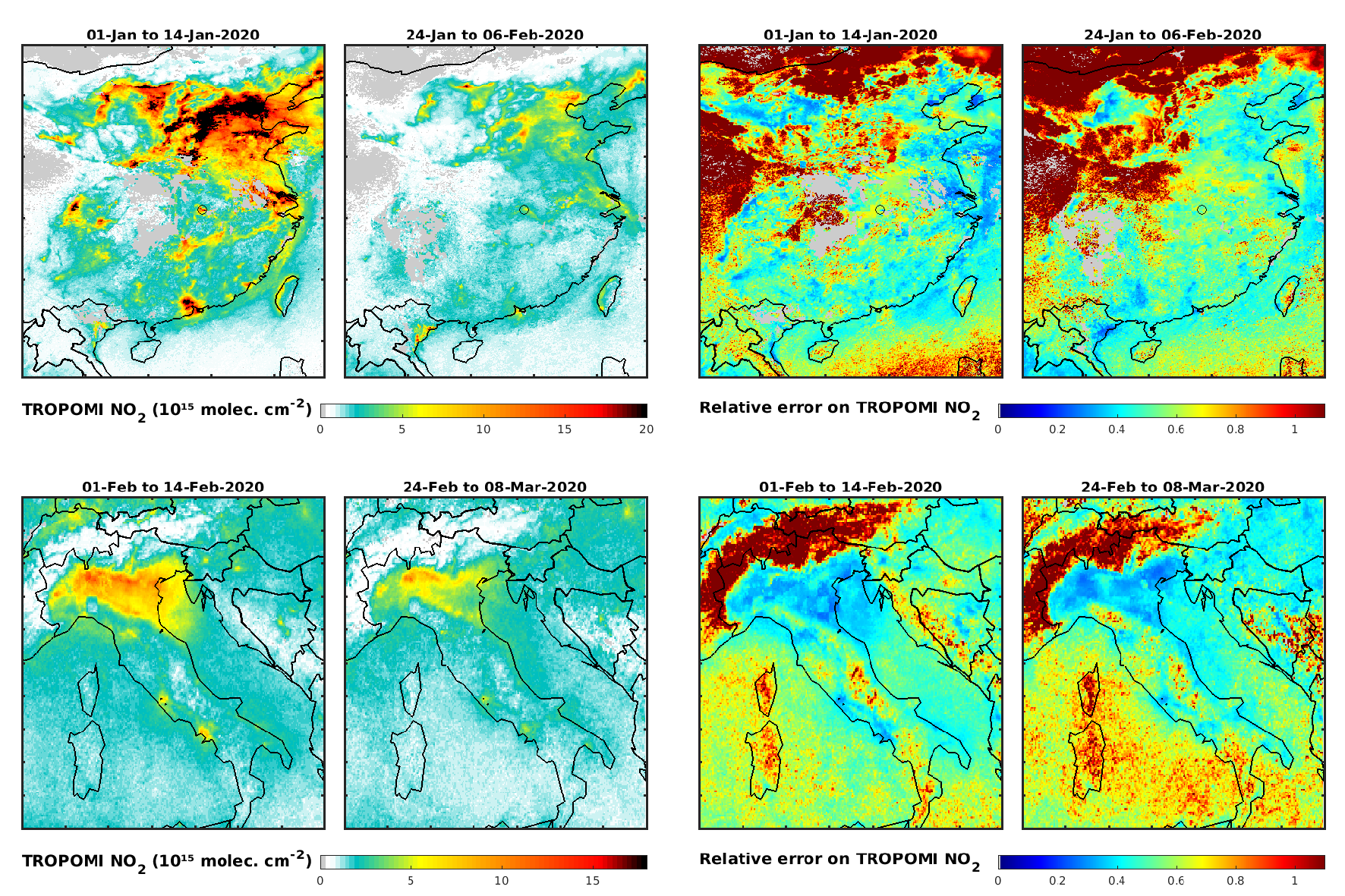


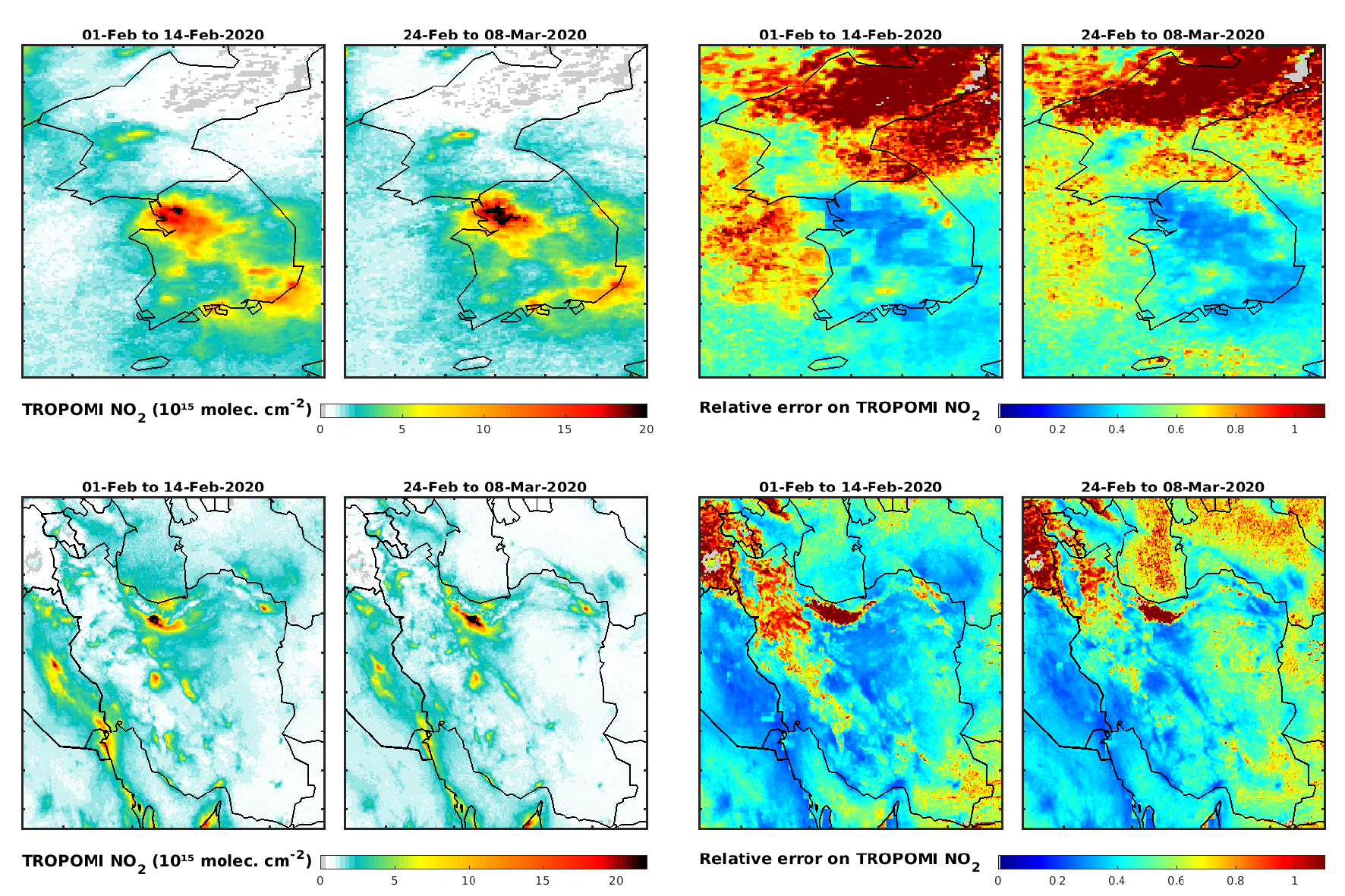


**Figure S8.** TROPOMI NO_2_ columns (left) and relative errors (right) in China, Italy, South Korea and Iran during the pre-lockdown and lockdown periods. The city of Wuhan is indicated by a open circle.
